# Tree-Based Machine Learning Models with Optuna in Predicting Impedance Values for Circuit Analysis

**DOI:** 10.3390/mi14020265

**Published:** 2023-01-20

**Authors:** Jung-Pin Lai, Ying-Lei Lin, Ho-Chuan Lin, Chih-Yuan Shih, Yu-Po Wang, Ping-Feng Pai

**Affiliations:** 1 PhD Program in Strategy and Development of Emerging Industries, National Chi Nan University, Puli Nantou 54561, Taiwan; 2 Siliconware Precision Industries Co., Ltd. No. 123, Sec. 3, Dafeng Rd., Dafeng Vil., Tanzi Dist., Taichung City 42749, Taiwan; 3 Department of Information Management, National Chi Nan University, Puli Nantou 54561, Taiwan

**Keywords:** integrated circuit, packaging and testing, machine learning, Optuna

## Abstract

The transmission characteristics of the printed circuit board (PCB) ensure signal integrity and support the entire circuit system, with impedance matching being critical in the design of high-speed PCB circuits. Because the factors affecting impedance are closely related to the PCB production process, circuit designers and manufacturers must work together to adjust the target impedance to maintain signal integrity. Five machine learning models, including decision tree (DT), random forest (RF), extreme gradient boosting (XGBoost), categorical boosting (CatBoost), and light gradient boosting machine (LightGBM), were used to forecast target impedance values. Furthermore, the Optuna algorithm is used to determine forecasting model hyperparameters. This study applied tree-based machine learning techniques with Optuna to predict impedance. The results revealed that five tree-based machine learning models with Optuna can generate satisfying forecasting accuracy in terms of three measurements, including mean absolute percentage error (*MAPE*), root mean square error (*RMSE*), and coefficient of determination (R2). Meanwhile, the LightGBM model with Optuna outperformed the other models. In addition, by using Optuna to tune the parameters of machine learning models, the accuracy of impedance matching can be increased. Thus, the results of this study suggest that the tree-based machine learning techniques with Optuna are a viable and promising alternative for predicting impedance values for circuit analysis.

## 1. Introduction

An integrated circuit (IC) comprises electronic circuits and components connected to each other via planar conductors that are electrically arranged on a planar silicon semiconductor substrate. Interconnections constitute the signal communication between the dies on a printed circuit board (PCB). Because signal integrity in high-speed circuit design is critical to electronic products, signal integrity issues have been essential for both high-speed circuit designers and PCB manufacturers. As a result, signal integrity has been extensively investigated in various high-speed and high-frequency applications [1,2,3,4]. Due to the close relationship between impedance and wiring patterns, impedance matching is one of the critical factors in high-speed PCB circuit design.

Impedance is the combination of capacitance and inductance in a high-frequency circuit. The controlled impedance in a printed circuit board ensures high signal integrity. The impedance *Z* is represented in Equation (1).

*Z* = *R* + j (*XL*–*XC*) (1)

where *R* is the resistance, j is the imaginary number, *XL* is the inductive reactance, and *XC* is the capacitive reactance. There are various signal transmissions in the wires on the circuit board. Preferably, the signal can be smoothly transmitted with minimal energy loss from the power supply to the receiving end. The receiving end then completely absorbs the energy without any reflection. However, the target impedance values slightly differ from the simulated values. The result of this difference causes impedance mismatch, overestimated reflections and losses, and poor signal integrity. Factors including line width, line spacing, dielectric thickness, the dielectric constant of the substrate, wire thickness, and surface roughness influence impedance. Incorrect single-ended or differential impedance causes signal reflection within the track when performing PCB layout. Thus, the loss of signal quality, lower operating frequency, and unwanted electromagnetic interference occur [5,6]. Therefore, controlling the circuit impedance within certain ranges when designing the PCB is critical in stabilizing PCB functions.

In recent years, emerging machine learning models and powerful data analysis techniques have been applied to various circuit design and analysis problems. Table 1 summarizes recent applications of machine learning approaches in circuit design and analysis. Zhang et al. [7] developed a deep neural network (DNN) to predict the impedance of a new board configuration. Their study revealed that well-trained DNNs performed 10,000 times faster than full-wave simulation methods. On the other hand, Juang et al. [8] employed genetic algorithms to decouple capacitor problems in a PCB power distribution network by selecting and placing capacitors to achieve the target impedance. Through the restriction of capacitors’ numbers, the designed approach can converge well and efficiently. The results indicated that computational effort increases as more calculations were performed, although more iterations could lead to a better solution.

Using decoupling capacitors, Xu et al. [9] presented a genetic algorithm-based method to optimize power delivery networks. The proposed method can also optimize jitter and power delivery networks (PDNs) impedance. According to the simulation and analysis results, the designed optimization method could reduce jitter and provide an optimal solution for the number of decoupling capacitors. Meanwhile, Park et al. [10] proposed an optimized decoupling capacitance design method based on a Q-learning algorithm for silicon interposer-based 2.5-D/3-D ICs. When testing power distribution networks, the presented approach was used to confirm target impedance values. The validation procedure was confirmed by comparing full-search simulations with the best result. The computation time of the proposed model was significantly less than that of the full-search simulation.

Swaminathan et al. [11] used machine learning techniques to solve signal and power integrity issues in package design. According to the finding of their study, using machine learning techniques logically can eliminate errors in the design process and thus, reduce design cycle time. Meanwhile, Cecchetti et al. [12] proposed an iterative optimization for the placement of decoupling capacitors in PDNs based on genetic algorithms (GA) and artificial neural networks (ANN). The study revealed that the designed GA-ANN model effectively produced results consistent with those obtained from the simulator generating a longer computation time.

Schierholz et al. [13] also used an ANN to predict target impedance violations in a large design space. The results of their study revealed that prediction accuracy in the design space for PDN impedance was very satisfactory. On the other hand, Zhang et al. [14] applied the deep reinforcement learning (DRL) approach and DNN to optimize the allocation of decoupling capacitors on priority positions. According to the results of their study, the proposed hybrid method could provide the minimum number of decoupling capacitors to satisfy the target impedance on a printed circuit board test.

Park et al. [15] created a DNN with regression and classification functions to conduct forecasting and classifying tasks for peak time-domain reflectometry impedance using silicon via void defects. Their study revealed that by partially tuning weights, the proposed models could provide accurate results. To estimate the impedance of power networks, Givaki et al. [16] proposed a random-forest model. The proposed model used the evolutionary multi-objective NSGA-II algorithm to adjust the random-forest model to estimate the resistance and inductive reactance accurately. In addition, Paulis et al. [17] employed genetic algorithms to optimize decoupling capacitors for PDN design at the PCB level to obtain a frequency spectrum at various locations. A close relationship between the measured results and the simulated input impedance revealed the effectiveness of the proposed method when validated on the board.

The characteristics of PCBs varies with different suppliers in the PCB circuit design and manufacturing process. The PCB foundry can precisely control the impedance characteristics when producing a PCB, while the signal transmission speed can be tested after the PCB board is manufactured. In this study, five tree-based machine learning models with the Optuna optimization algorithm were used to forecast the target impedance values. Optuna was used to determine the hyperparameters of machine learning models. The rest of this study is organized as follows. Section 2 depicts the PCB-based substrate and circuit transfer characteristics. Section 3 introduces the machine learning models and the Optuna optimization algorithm, while Section 4 and Section 5 describe the numerical results and conclusion, respectively.

## 2. IC-PCB Circuit Signal Transmission and Substrate Structure

Impedance matching is a common working state in PCB circuits, reflecting the power transfer relationship between the input and the output circuits. PCB or substrate design is responsible for the characteristic impedance discontinuities of interconnections for signal integrity. Maximum power transfer, on the other hand, is achieved when the circuit impedance is matched. Signal integrity and power loss are influenced by the impedance gaps between the IC package and the PCB system. Reflections can cause unexpected noises in systems [18].

**Figure 1:**
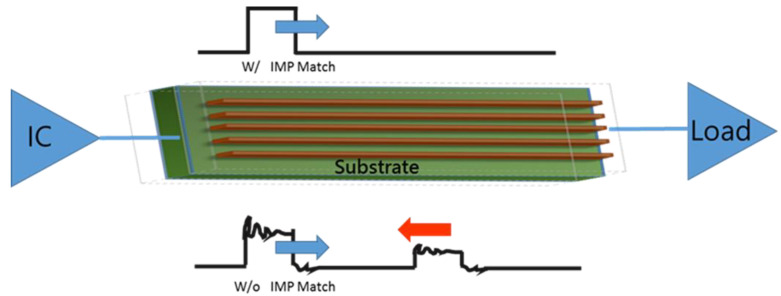
Circuit signal transmission on the substrate.

Figure 1 shows the circuit signal transmission on the substrate. As depicted in Figure 1, the impedance gaps have a significant impact on signal integrity and power loss. The internal impedance of the signal transmitter should ideally be the same as the target impedance of the transmission line at the source to reduce reflections when sending signals. Meanwhile, to communicate the signal between the chip and the circuit board, the circuit inside the IC carrier board connects the chip and the external circuit board together. Lines and drawings, dielectric layers, holes, and solder resist ink make up the substrate.

**Figure 2:**
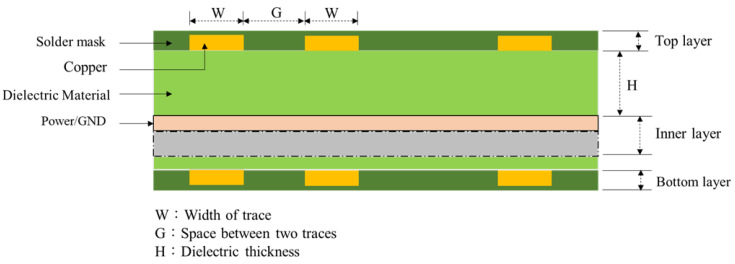
Multilayer PCB stack-up.

Figure 2 depicts a multilayer PCB stack-up. When using a time-domain reflectometer to measure impedance signals, probes are used for the outer signal line and GND pin. The measurement includes the metal and dielectric layers in the inner layer of the PCB stack-up.

Since the circuit performance of the PCB board provided must be able to ensure the signal is not reflected during the transmission process, which keeps the signal intact and reduces the transmission loss, this plays a crucial role in the substrate material of impedance [19,20].

## 3. Tree-Based Machine Learning Architecture to Predict Impedance Value

**Figure 3:**
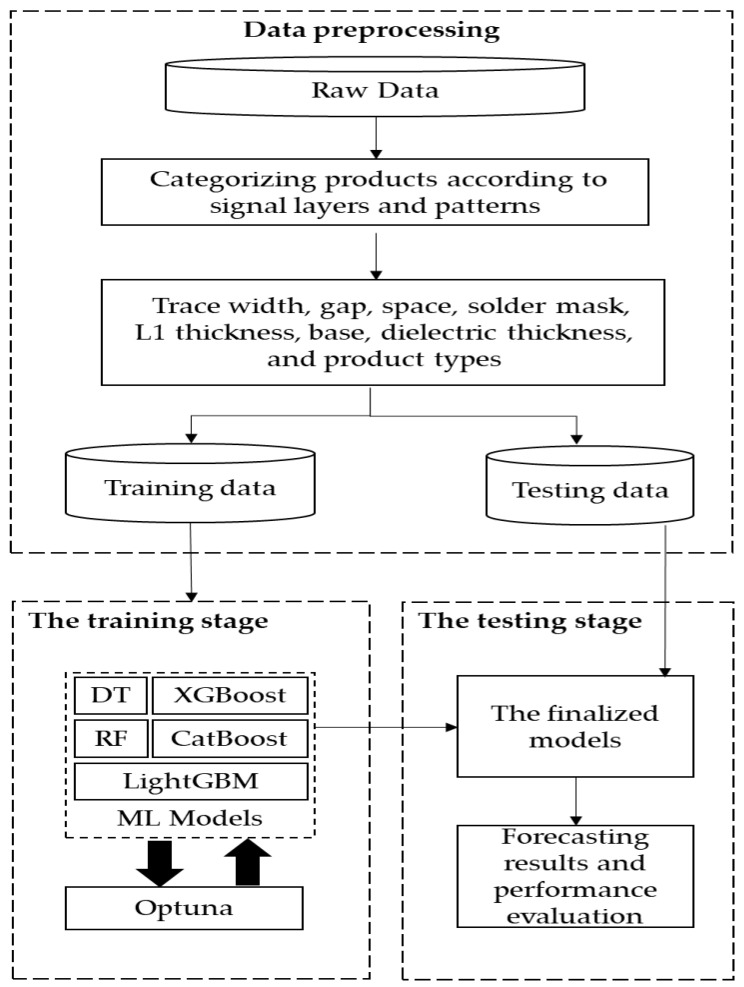
The architecture of forecasting impedance values by tree-based machine learning models with Optuna.

Figure 3 illustrates the proposed architecture for impedance value prediction. As shown in Figure 3, the architecture was divided into three stages: data preprocessing, training stage, and testing stage. SPIL (Siliconware Precision Industries Co., Ltd., Taichung, Taiwan) provided the raw impedance data used in this study. Each dataset was preprocessed and divided into 80% training data and 20% testing data. Training data was employed to build models with tree-based machine-learning (ML) methods during the training stage. Five models, including decision tree (DT), random forest (RF), extreme gradient boosting (XGBoost), categorical boosting (CatBoost), and light gradient boosting machine (LightGBM) were used in this study. In addition, the Optuna framework was used to determine model hyperparameters. Finally, testing data was used to predict the finalized model, and the forecasting performances were evaluated.

### 3.1. Data Preprocessing

Table 2 presents the PCB products’ raw data, including product types and variables of impedance. The PCB products’ raw data were categorized into different datasets based on the following attributes: signal layers and patterns. Seven attributes were used as independent variables in this study. These attributes include trace width, gap, space, solder mask, L1 thickness, base, and dielectric thickness. According to the manufacturing process, the data of products were classified into three categories represented by GSSG, SS, and S for category A, B, and C, respectively. The signal layer has different layers based on PCB layer structures. Table 3 displays the datasets for product subcategories based on the signal layers and patterns.

### 3.2. Tree-Based Machine Learning

Tree-based machine learning models are defined as supervised machine learning algorithms employed for solving problems of classification and regression. In the tree-dividing procedure, the training data was divided into subsets, where every split increases the complexity of models to conduct the task well [21,22,23,24,25].

In addition to the basic decision tree (DT) and the random forest (RF), the extreme gradient boosting (XGBoost), light gradient-boosting machine (LightGBM), and the categorical boosting (CatBoost) are popular and powerful methods with outstanding performance in many fields. DT and RF are basic tree-based machine learning, while XGBoost, CatBoost, and LightGBM are advanced models of gradient-boosting decision trees. Tree-based machine-learning models have been used in many fields, such as economics and finance [26,27], politics [28], business and insurance [29,30], biology and environment [31,32], and medicine and healthcare [33,34]. However, the applications of tree-based machine-learning models in forecasting impedance values for circuit analysis have not been widely investigated. Thus, this study used Optuna to determine hyperparameters for tree-based machine learning models, applied to impedance values for the PCB industry.

The first tree-based model used in this study is the decision tree. As one of the basic methods for dealing with regression and classification problems [35], the decision tree conducts regression and classification tasks by variables with continuous and discrete values, respectively [36]. This study used decision trees for regression problems. Table 4 indicates the hyperparameters and search ranges of the decision tree model used in this study [37,38,39].

The second technique employed in this study is the random forest. Developed by Breiman [40], the random forecast is composed of multiple decision trees and performs random feature selection of each tree, then averages output values of all individual trees to obtain the model’s output [41]. Table 5 depicts the hyperparameters and searching ranges used in the random forecast method. As shown in Table 5, hyperparameters contain the number of trees in the forest (n_estimators), the max number of levels in each decision tree (max_depth), and the number of data points placed in a node before the node is split (min_samples_split) [37,38,39,42].

XGBoost [43] approach is the third tree-based machine learning model employed in this study. The XGBoost combines two characteristics, bagging and boosting, for ensemble learning. The bagging trains models in parallel and generates trees by independent sampling. The advantage of bagging policy is to increase the stability and accuracy of models. The boosting generates trees sequentially, and each tree is related to each other. The generation of each tree in the boosting procedure can improve the poor learning of the previous tree [44,45]. Table 6 presents the hyperparameters and the search ranges of the XGBoost model in this study [46,47].

Sequentially, the CatBoost [48], which is one of the gradient-boosting algorithms based on decision trees, was introduced in this study. By using an ensemble learning strategy, the CatBoost approach takes advantage of the combination of weaker regression models to form a robust regression model. Table 7 illustrates hyperparameters and the search ranges of the CatBoost model in this study [47,49,50,51].

Lastly, this study employed the LightGBM to forecast the impedance values for circuit analysis. The LightGBM is a lightweight algorithm based on the gradient-boosting algorithm proposed by Ke et al. [52]. LightGBM approach uses a novel, gradient-based, and one-sided sampling technique to filter data instances and generate segmentation values. In addition, exclusive feature bundling is conducted to reduce the number of features. Thus, the LightGBM results in an efficient training procedure. Table 8 shows hyperparameters and the search ranges of the LightGBM model in this study [26,53,54,55,56].

Finally, the LightGBM was employed in this study to forecast the impedance values for circuit analysis. The LightGBM is a lightweight algorithm based on gradient boosting proposed by Ke et al., [52]. LightGBM approach uses a novel gradient-based one-sided sampling technique to filter data instances and generate segmentation values. In addition, the exclusive feature bundling is conducted to reduce the number of features. Thus, the LightGBM results in an efficient training procedure. Table 8 depicts hyperparameters and the searching ranges of the LightGBM model in this study [26,53,54,55,56].

### 3.3. Optuna for Selecting Hyperparameters of Tree-Based Machine Learning Models

Determining hyperparameters for tree-based machine learning models significantly influences the forecasting performance [57,58].

Optuna [59] is an emerging tool with three advantages for model selection or hyperparameters determination. The first advantage Optuna provides is the define-by-run style API. The second advantage is an efficient pruning and sampling mechanism. The third advantage is that it is easy to set up. The concept of define-by-run style API comes from a deep-learning framework. It enables users to decide the hyperparameter search space dynamically. Meanwhile, two efficient sampling and pruning mechanism policies are efficient searching and efficient performance estimation, both of which request the cost-effective optimization method. On the other hand, the most commonly used sampling methods are relational sampling and independent sampling, represented by covariance matrix adaptation evolution strategy (CMA-ES) and (tree-structured Parzen estimator) TPE, respectively. Specifically, Optuna allows customized sampling procedures. In terms of the pruning mechanism, two phases were performed. First, the intermediate objective values were periodically monitored. Second, the trail is terminated when the predefined condition is not met. Optuna’s last design feature is associated with its ease of setup, which allows it to be easily configured for lightweight experiments to heavy-weight distributed computations under the versatile architecture [60,61].

**Figure 4:**
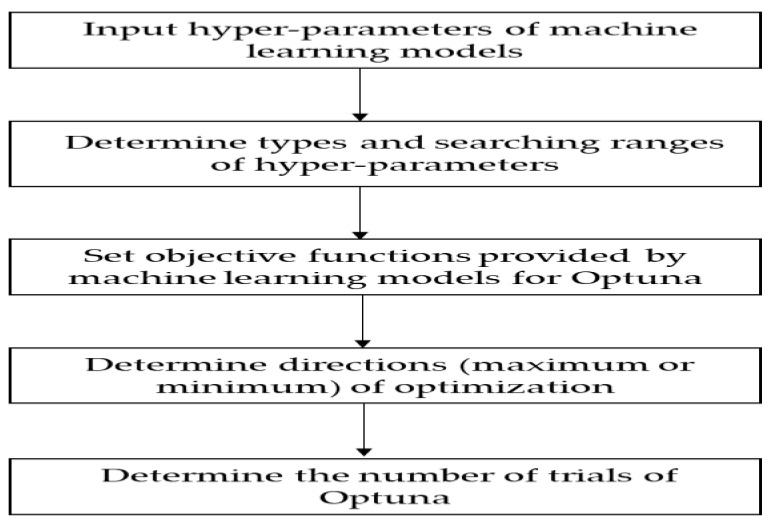
The basic Optuna procedure for hyperparameters selection.

Figure 4 depicts the essential steps in determining hyperparameters for machine learning models in Optuna. The first step is to enter the hyperparameters of machine learning models. In this study, five tree-based machine learning models, each having a different set of hyperparameters, were used. The second step is determining the search ranges of hyperparameters and types, including integers, real numbers, and categorical numbers. The third step is to set the objective function for Optuna, as provided by the machine learning models. Then, optimization directions are determined. Minimizing forecasting errors serves as the direction and the objective function of this study. Finally, the number of trials of Optuna is set. In this research, the sampler, direction, and n_trials are set to TPE sampler, minimum, and 100, respectively.

## 4. Numerical Results

This study demonstrated five tree-based ML methods: DT, FR, XGBoost, CatBoost, and LightGBM. To predict the impedance value, this study optimized each model’s hyperparameters using Optuna. Three evaluation metrics were used to evaluate the experimental results of this study: mean absolute percentage error (*MAPE*), root mean square error (*RMSE*), and coefficient of determination (R2). As shown in Equations (2)–(4).

(2) $$MAPE\left(\%\right)=\frac{100}{n}\overset{n}{\underset{i=1}{\sum }}\left|\frac{Y_{i}-{\hat{Y}}_{i}}{Y_{i}}\right|$$

(3) $$RMSE=\sqrt{\frac{\Sigma_{i=1}^{n}{\left(Y_{i}-{\hat{Y}}_{i}\right)}^{2}}{n}}$$

(4) $$R2=1-\frac{\Sigma_{i=1}^{n}{\left(Y_{i}-{\hat{Y}}_{i}\right)}^{2}}{\Sigma_{i=1}^{n}{\left(Y_{i}-\bar{Y}\right)}^{2}}$$

where n is the number of forecasting instance, $Y_{i}$ is the $i^{\mathrm{th}}$ actual impedance value, ${\hat{Y}}_{i}$ is the $i^{\mathrm{th}}$ forecasting impedance value, and $\bar{Y}$ is the mean value of actual impedance value.

**Figure 5:**
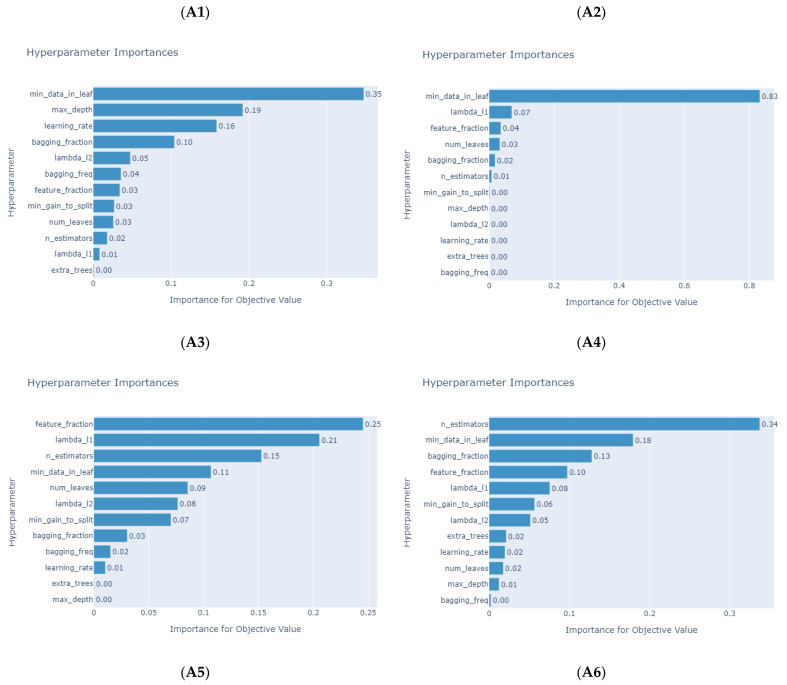

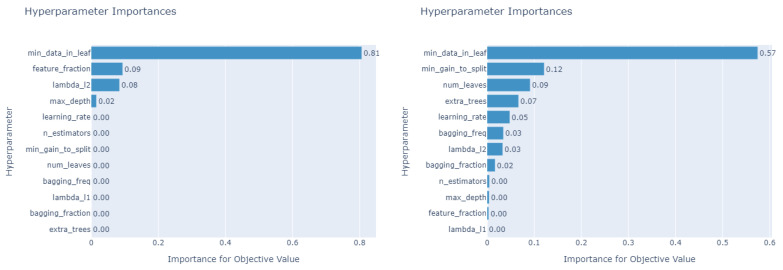
Hyperparameter importance of LightGBM models for product category A (product **A1**–**A6**).

**Figure 6:**
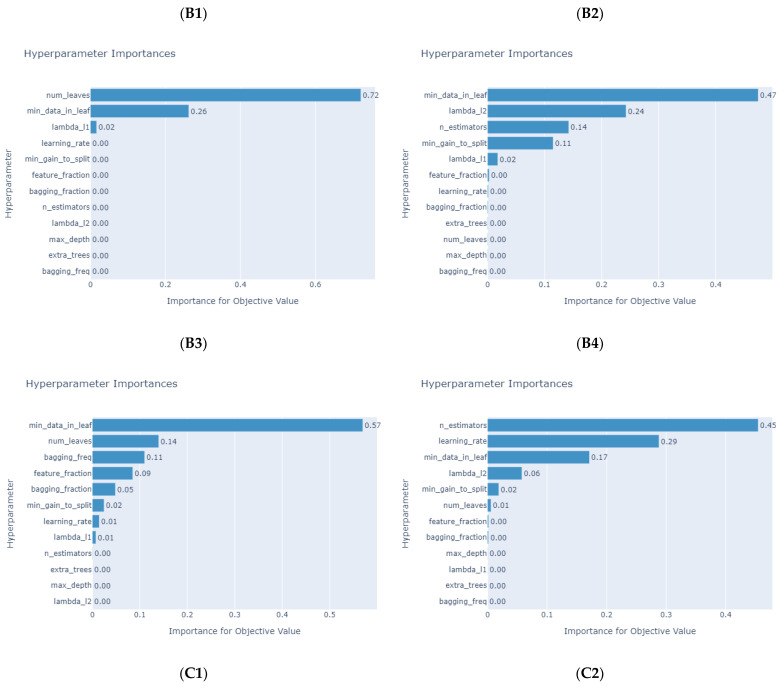

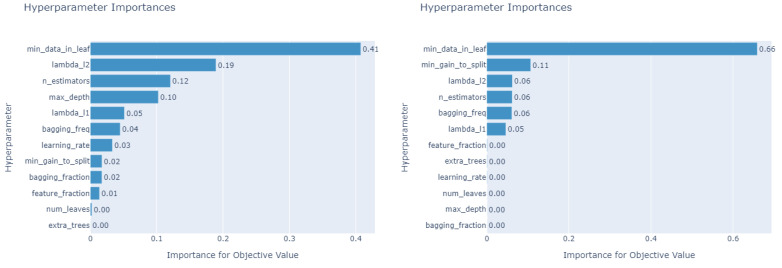
Hyperparameter importance of LightGBM models for product categories B and C (product **B1**–**B4**, **C1**–**C2**).

**Figure 7:**
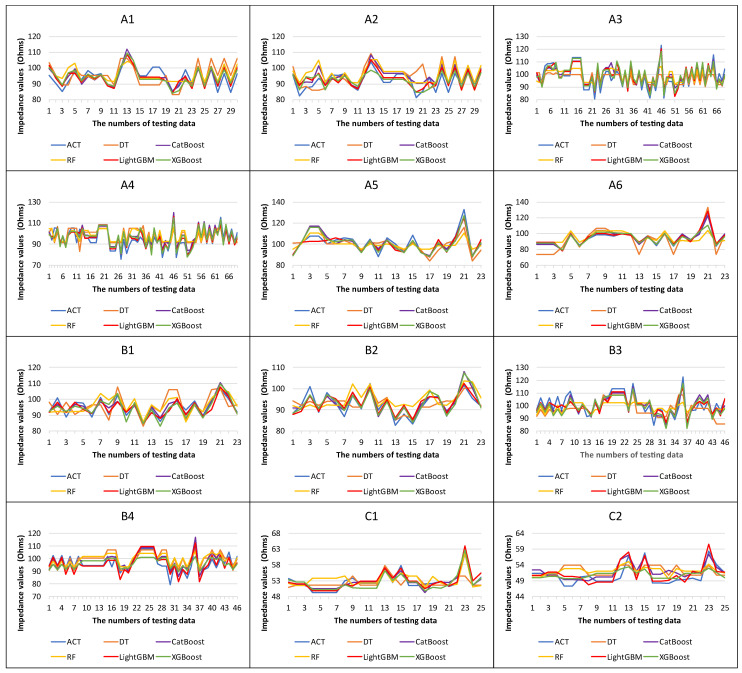
The point-to-point plots of actual and predicted values of impedance for five machine learning models (product **A1**–**A6**, **B1**–**B4**, **C1**–**C2**).

Table 9 and Table 10 depict the hyperparameters determined by Optuna. Figure 5 and Figure 6 present the importance of the LightGBM models’ hyperparameters for different products, indicating that the hyperparameter of the “min_data_in_leafe” is the most important in most products. Table 11 illustrates the prediction results of the tree-based machine learning models. Overall, the average *MAPE* and *RMSE* of impedance-predicting results are low. LightGBM has the best performance in all datasets, followed by XGBoost and CatBoost, while DT and RF performances are slightly inferior. In addition, five tree-based machine learning models with Optuna can obtain *MAPE* values less than 10 and can be treated as accurate forecasting models [62]. R2 is the measurement of the independent variables’ abilities to interpret dependent variables. When the R2 value is close to 1, the explanatory abilities of independent variables are at higher levels [63,64,65]. Among all models, the most explanatory is LightGBM, followed by XGBoost and CatBoost, and finally DT and RF. Figure 7 provides the actual and predicted values of impedance for the five tree-based machine learning models used in this study. Thus, the proposed tree-based machine learning models are useful and can be duplicated in forecasting impedances accurately during the design process of PCB. Therefore, the PCB design time can be reduced effectively.

## 5. Conclusions

This study used five tree-based machine-learning techniques with Optuna to predict impedance due to the differences between the circuit simulation and the actual measurement in the production process of PCB wiring impedance. The forecasting outcomes revealed that tree-based machine learning models with Optuna are feasible and accurate methods for predicting target impedance values. The light gradient-boosting machine with Optuna performed the best in three forecasting measurements. Thus, the proposed tree-based machine learning using the Optuna model is useful when defining target impedances during design, simulation, and manufacturing, as it improves the impedance prediction for PCB designers and manufacturers. For the current practical manufacturing process, manufacturers can use the existing impedance data and perform accurate impedance prediction through the method proposed in this research to shorten the PCB design and process time. Future studies may employ more impedance value forecasting cases to examine the robustness of the designed machine learning techniques in predicting the target impedance. The other potential direction for future work is applying other forecasting techniques to obtain more accurate results.

## Figures and Tables

**Table 1:** Recent applications of machine learning approaches in circuit design and analysis.

Literature	Years	Applications	Methods
Zhang et al. [7]	2022	Impedance prediction	DNN
Juang et al. [8]	2022	Decoupling capacitor	Genetic Algorithms
Xu et al. [9]	2021	Decoupling placement optimization	Genetic Algorithms
Park et al. [10]	2020	Decoupling capacitor	Q-Learning
Swaminathan et al. [11]	2020	Signal and power integrity	FFNN, RNN, CNN
Cecchetti et al. [12]	2020	Decoupling capacitor	GA-ANN
Schierholz et al. [13]	2020	Predicting target impedance violations	ANN
Zhang et al. [14]	2019	Decoupling capacitor	DRL, DNN
Park et al. [15]	2019	Signal and power integrity	DNN
Givaki et al. [16]	2019	Impedance estimation	Random Forest
Paulis et al. [17]	2019	Decoupling placement optimization	Genetic Algorithms

**Table 2:** The corresponding attributes according to patterns’ categories.

Product Categories	Patterns	Attributes (Variables)						
		X1	X2	X3	X4	X5	X6	X7
		Trace Width (um)	Gap (um)	Space (um)	Solder Mask (um)	L1 Thickness (um)	Base (um)	DielectricThickness (um)
A	GSSG	X	X	X	X	X	X	X
B	SS	X	X		X	X	X	X
C	S	X			X	X	X	X

**Table 3:** The datasets for product subcategories based on the signal layers and patterns.

Product Subcategories	Signal Layers	Patterns	Instances
A1	Base	GSSG	146
A2	L1	GSSG	146
A3	Base	GSSG	343
A4	L1	GSSG	342
A5	Base	GSSG	114
A6	L1	GSSG	113
B1	Base	SS	115
B2	L1	SS	115
B3	Base	SS	229
B4	L1	SS	228
C1	Base	S	122
C2	L1	S	122

**Table 4:** Hyperparameters of the decision tree model tuned in this study [37,38,39].

Hyperparameters	Implications	Types	Search Ranges
splitter	The strategy used for choosing the division.	Categorical numbers	‘best’, ‘random’
max_depth	The maximum depth of the tree.	Integers	2, 24
min_samples_split	The minimum number of samples required to split an internal node.	Integers	2, 9
max_features	The number of features to consider when looking for the best split	Categorical numbers	‘sqrt’, ‘auto’, ‘log2’
max_leaf_nodes	Grow a tree with max_leaf_nodes in best-first fashion.	Integers	10, 1000

**Table 5:** Hyperparameters of the random forest model tuned in this study [37,38,39,42].

Hyperparameters	Implications	Types	Search Ranges
n_estimators	The number of trees in the forest.	Integers	50, 1000
max_depth	The maximum depth of the tree.	Integers	2, 24
min_samples_split	The minimum number of data points in a node before the node is split.	Integers	2, 9

**Table 6:** The main hyperparameters of the XGBoost model tuned in this study [46,47].

Hyper-Parameter	Implication	Types	Search Ranges
lambda	L2 regularization term on weights.	Real numbers	0.00001, 10
alpha	L1 regularization term on weights.	Real numbers	0.00001, 10
colsample_bytree	The subsample ratio of columns when constructing each tree.	Real numbers	0.2, 0.6
subsample	The subsample ratio of the training instances.	Real numbers	0.4, 0.8
learning_rate	The learning rate.	Real numbers	0.0001, 0.2
n_estimators	The number of trees.	Integers	50, 10,000
max_depth	The maximum depth of a tree.	Integers	2, 12
min_child_weight	The minimum sum of instance weight (hessian) needed in a child.	Integers	1, 300

**Table 7:** Hyperparameters of the CatBoost model tuned in this study [47,49,50,51].

Hyper-Parameter	Implication	Types	Search Ranges
iterations	The maximum number of trees.	Integers	50, 10,000
depth	The maximum depth of the tree.	Integers	2, 12
learning_rate	The learning rate.	Real numbers	0.0001, 0.2
l2_leaf_reg	Coefficient at the L2 regularization term of the cost function.	Real numbers	0.00001, 10
bagging_temperature	Defines the settings of the Bayesian bootstrap.	Real numbers	0.01, 10
min_child_samples (min_data_in_leaf)	The minimum number of training samples in a leaf.	Integers	5, 100

**Table 8:** Hyperparameters of the LightGBM model tuned in this study [26,53,54,55,56].

Hyperparameters	Implications	Types	Search Ranges
n_estimators	The number of trees.	Integers	50, 10,000
learning_rate	The learning rate.	Real numbers	0.0001, 0.2
num_leaves	The number of leaves per tree.	Integers	2, 2048
max_depth	The maximum learning depth.	Integers	2, 12
min_data_in_leaf	The minimal number of data in one leaf. Prevent overfitting.	Integers	1, 100
lambda_l1	L1 regularization. Prevent overfitting.	Real numbers	0.00001, 10
lambda_l2	L2 regularization. Prevent overfitting.	Real numbers	0.00001, 10
min_gain_to_split	The minimal error reduction to conduct the further split.	Real numbers	0, 15
bagging_fraction	The ratio of the selected data to the total data.	Real numbers	0.3, 1.0
bagging_freq	Frequency of re-sampling the data when bagging_fraction is smaller than 1.0.	Integers	1, 7
feature_fraction	The proportion of the selected feature to the total number of features.	Real numbers	0.3, 1.0
extra_trees	Uses randomized trees.	Categorical numbers	‘True’, ‘False’

**Table 9:** The hyperparameters for category A models provided by Optuna.

Methods	Hyperparameters	A1	A2	A3	A4	A5	A6
DT	splitter	random	random	random	random	random	random
	max_depth	5	19	3	3	11	15
	min_samples_split	8	3	8	3	9	5
	max_features	sqrt	log2	log2	log2	sqrt	sqrt
	max_leaf_nodes	730	886	604	359	279	778
	FrozenTrial ^#^	41	31	85	45	77	21
FR	n_estimators	50	273	140	184	328	60
	max_depth	2	2	2	2	2	2
	min_samples_split	5	4	2	3	9	9
	FrozenTrial ^#^	76	53	13	60	93	12
XGBoost	lambda	0.00013	9.99880	7.39058	0.03166	0.33494	0.00003
	alpha	0.17368	0.11708	0.00006	4.42219	0.31300	1.30132
	colsample_bytree	0.53136	0.43740	0.47339	0.50856	0.59988	0.31599
	subsample	0.72083	0.68340	0.58809	0.71827	0.50646	0.72486
	learning_rate	0.02817	0.02235	0.16934	0.03755	0.00503	0.00407
	n_estimators	3502	8701	3107	1555	3314	7162
	max_depth	3	4	2	10	3	4
	min_child_weight	3	1	1	5	1	1
	FrozenTrial ^#^	27	80	96	14	87	52
CatBoost	iterations	1361	1428	4808	7459	5246	9659
	depth	2	11	4	10	4	7
	learning_rate	0.16536	0.02557	0.14240	0.00447	0.01723	0.01481
	l2_leaf_reg	0.00031	0.00052	0.00003	0.00037	3.60489	0.00001
	bagging_temperature	0.01262	3.30881	0.06255	1.54283	3.77145	0.06189
	min_child_samples	48	25	29	45	49	49
	FrozenTrial ^#^	94	14	72	5	82	57
LightGBM	n_estimators	6775	7866	4876	8457	7635	1355
	learning_rate	0.12735	0.19625	0.17452	0.13543	0.16941	0.18596
	num_leaves	649	1631	1599	517	19	1150
	max_depth	12	8	10	6	7	4
	min_data_in_leaf	6	10	1	6	3	3
	lambda_l1	0.65465	0.40568	0.10117	4.44552	0.01356	0.30749
	lambda_l2	0.02067	7.10369	0.00002	3.64057	0.00007	0.00032
	min_gain_to_split	0.62324	6.27989	8.02722	0.01769	0.51251	3.25957
	bagging_fraction	0.71382	0.66880	0.55024	0.78785	0.73125	0.84466
	bagging_freq	5	1	3	7	3	2
	feature_fraction	0.97166	0.36366	0.84806	0.81182	0.79440	0.57880
	extra_trees	TRUE	FALSE	TRUE	FALSE	TRUE	FALSE
	FrozenTrial ^#^	41	82	71	30	82	92

^#^: The consecutive number of trial for each Study of Optuna

**Table 10:** The hyperparameters for category B and C models provided by Optuna.

Methods	Hyperparameters	B1	B2	B3	B4	C5	C6
DT	splitter	random	best	Best	random	random	best
	max_depth	17	8	14	3	2	2
	min_samples_split	3	2	9	6	7	3
	max_features	sqrt	log2	log2	sqrt	sqrt	log2
	max_leaf_nodes	371	522	280	228	894	41
	FrozenTrial ^#^	19	40	96	78	10	70
FR	n_estimators	107	115	273	410	609	66
	max_depth	2	2	2	2	2	2
	min_samples_split	5	9	5	2	8	6
	FrozenTrial ^#^	45	11	99	98	85	15
XGBoost	lambda	0.10002	0.02107	0.35943	0.00057	0.00014	3.33129
	alpha	0.00025	0.02653	0.01639	5.60999	0.48286	0.01335
	colsample_bytree	0.57253	0.53535	0.35838	0.23654	0.53890	0.53090
	subsample	0.58380	0.72630	0.40557	0.79891	0.40926	0.69241
	learning_rate	0.02778	0.02027	0.16094	0.02471	0.00655	0.01433
	n_estimators	2002	2416	2905	9578	5672	2965
	max_depth	12	3	2	10	5	7
	min_child_weight	1	1	1	2	3	8
	FrozenTrial ^#^	78	99	78	48	6	91
CatBoost	iterations	5168	8064	5747	3031	2759	7897
	depth	11	4	4	11	6	8
	learning_rate	0.01978	0.02600	0.12381	0.02291	0.00251	0.06970
	l2_leaf_reg	0.00001	0.18489	0.00003	0.07952	0.00251	0.00017
	bagging_temperature	1.11136	0.13162	0.62528	0.22679	0.23521	1.79410
	min_child_samples	77	74	49	81	89	65
	FrozenTrial ^#^	90	87	91	23	17	92
LightGBM	n_estimators	4130	2450	8692	8417	5671	3523
	learning_rate	0.12182	0.17747	0.17526	0.19914	0.16131	0.18101
	num_leaves	670	924	1757	153	207	1683
	max_depth	12	5	9	10	3	3
	min_data_in_leaf	1	1	12	3	9	2
	lambda_l1	0.00268	0.00543	0.02561	0.13634	0.00013	0.20529
	lambda_l2	0.01191	0.23902	0.11103	0.02720	0.00183	0.00008
	min_gain_to_split	4.40312	1.28234	0.03857	4.66666	5.22525	0.41965
	bagging_fraction	0.40501	0.95348	0.84419	0.91639	0.64485	0.41063
	bagging_freq	7	2	1	6	1	5
	feature_fraction	0.77680	0.49448	0.89466	0.61959	0.92896	0.90923
	extra_trees	FALSE	FALSE	FALSE	TRUE	FALSE	TRUE
	FrozenTrial ^#^	24	29	61	19	56	99

^#^: The consecutive number of trial for each Study of Optuna

**Table 11:** The *RMSE* and R2 results of the tree-based ML model prediction.

Dataset	DT	RF	XGB	CatBoost	LightGBM	Average
	with Optuna					
***MAPE***						
A1	5.36%	4.38%	3.19%	2.77%	2.74%	3.68%
A2	5.42%	5.17%	3.32%	3.63%	3.12%	4.13%
A3	4.64%	4.80%	3.25%	3.64%	3.25%	3.92%
A4	6.89%	6.45%	3.85%	4.15%	3.46%	4.96%
A5	5.36%	5.36%	2.59%	3.10%	2.20%	3.72%
A6	6.25%	5.27%	1.81%	1.77%	1.38%	3.29%
B1	5.38%	4.11%	2.59%	2.16%	1.99%	3.25%
B2	3.27%	4.48%	2.18%	2.37%	1.99%	2.86%
B3	6.04%	5.30%	3.58%	3.33%	2.99%	4.25%
B4	5.31%	5.87%	4.88%	3.72%	2.67%	4.49%
C1	3.54%	3.38%	2.31%	1.86%	1.69%	2.56%
C2	5.40%	5.41%	3.99%	3.49%	2.67%	4.19%
Average	5.24%	5.00%	3.13%	3.00%	2.51%	3.78%
***RMSE***						
A1	6.12	4.79	3.64	3.19	3.17	4.18
A2	6.44	5.73	3.53	4.20	3.31	4.64
A3	6.34	5.73	3.67	4.23	3.55	4.71
A4	8.05	7.27	4.39	4.60	4.09	5.68
A5	6.94	6.99	3.59	4.35	3.20	5.02
A6	7.58	6.89	3.86	2.23	1.45	4.40
B1	5.88	4.65	2.94	2.40	2.36	3.65
B2	3.84	4.86	2.67	2.66	2.21	3.25
B3	7.40	7.00	4.31	4.10	3.84	5.33
B4	6.05	6.56	5.39	4.22	3.28	5.10
C1	2.53	2.32	1.46	1.16	1.11	1.72
C2	3.36	3.13	2.39	2.13	1.90	2.58
Average	5.88	5.49	3.49	3.29	2.79	4.19
**R2**						
A1	−0.12	0.32	0.60	0.70	0.70	0.44
A2	−0.39	−0.10	0.58	0.41	0.63	0.23
A3	0.46	0.56	0.82	0.76	0.83	0.69
A4	0.17	0.33	0.75	0.73	0.79	0.55
A5	0.48	0.48	0.86	0.80	0.89	0.70
A6	0.37	0.48	0.84	0.95	0.98	0.72
B1	−0.12	0.30	0.72	0.81	0.82	0.51
B2	0.49	0.19	0.75	0.76	0.83	0.60
B3	0.24	0.32	0.74	0.77	0.80	0.57
B4	0.25	0.11	0.40	0.63	0.78	0.44
C1	0.12	0.26	0.71	0.81	0.83	0.55
C2	−0.28	−0.11	0.35	0.49	0.59	0.21
Average	0.14	0.26	0.68	0.72	0.79	0.52

## Data Availability

Not applicable.
